# Immunology of allergen immunotherapy

**DOI:** 10.1093/immadv/ltac022

**Published:** 2022-11-25

**Authors:** Rifat S Rahman, Duane R Wesemann

**Affiliations:** Harvard Medical School, Boston, MA, USA; Department of Medicine, Division of Allergy and Clinical Immunology, Division of Genetics, Brigham and Women’s Hospital, Harvard Medical School, Boston, MA, USA; Ragon Institute of MGH, MIT, and Harvard, Boston, MA, USA; Broad Institute of MIT and Harvard, Boston, MA, USA

**Keywords:** epicutaneous immunotherapy, intradermal immunotherapy, intralymphatic immunotherapy

## Abstract

Allergen immunotherapy (AIT) is the only disease-modifying therapy for allergic disease. Through repeated inoculations of low doses of allergen—either as whole proteins or peptides—patients can achieve a homeostatic balance between inflammatory effectors induced and/or associated with allergen contact, and mediators of immunologic non-responsiveness, potentially leading to sustained clinical improvements. AIT for airborne/respiratory tract allergens and insect venoms have traditionally been supplied subcutaneously, but other routes and modalities of administration can also be effective. Despite differences of allergen administration, there are some similarities of immunologic responses across platforms, with a general theme involving the restructuring and polarization of adaptive and innate immune effector cells. Here we review the immunology of AIT across various delivery platforms, including subcutaneous, sublingual, epicutaneous, intradermal, and intralymphatic approaches, emphasizing shared mechanisms associated with achieving immunologic non-responsiveness to allergen.

## Introduction

Allergic disease is a growing global problem driven by a pathophysiology that is not yet fully understood. Allergen immunotherapy (AIT) is a process of supplying the allergen to which the patient is sensitized (i.e. allergic) in small doses, escalating over time until reaching a maintenance dose that continues to be provided for an extended period. While patients are receiving AIT, they may become ‘desensitized’ to the allergen; that is, they become transiently non-responsive upon exposure to allergen [1]. The ideal outcome of AIT is to achieve a state of sustained non-responsiveness that may persist even after discontinuation of therapy, reflecting a persistent restructuring of the immune system; however, this has only been demonstrated in a few instances.

Allergens used in AIT have traditionally been aeroallergens, such as plant pollens, dust, and animal dander, as well as insect venom allergens, supplied via the subcutaneous route. Other routes of entry have been studied as well, particularly for food allergens via the direct oral route. Different routes of AIT also include sublingual, epicutaneous, intradermal, and intralymphatic.

Mechanisms of allergic sensitization in humans remain largely mysterious, in part because it often occurs at some undetermined time and place before the onset of symptoms. While it is unlikely that desensitization is simply the reverse of sensitization, examination of how different routes of allergen administration influences response may provide some hints into long-standing questions about how sensitized states are established and maintained. In addition, understanding how route of administration influences AIT outcomes could shed light on optimal methods for achieving immunologic non-responsiveness and provide practical options for patient care.

## Pathophysiology of allergic inflammation

In allergic disease, various allergens including pollens, dust mites, animal dander, insect venoms, and foods trigger an IgE-mediated inflammatory response [2]. The route of exposure, such as inhalation in the case of aeroallergens or skin contact or consumption in the case of oral allergens, determine which tissues initially encounter the allergen and influence how symptoms manifest.

Allergic inflammation relies on an initial sensitization event whereby allergens enter and are subsequently taken up by local antigen-presenting cells (APCs) which migrate to regional lymph nodes (LNs) and activate CD4^+^ T lymphocytes to elicit a T helper cell (Th) type 2-response [3, 4]. In Th2 inflammation, IL-4 and IL-13 stimulate B cell class switching to IgE in a STAT6-dependent mechanism [5, 6]. Following initial sensitization, re-exposure of allergen can induce allergic inflammation. Within seconds to minutes, allergens interact with and crosslink the high-affinity IgE receptor (FcεRI), initiating a signaling cascade leading to the degranulation of mast cells and basophils with release of histamines, leukotrienes, and prostaglandins [7, 8]. These inflammatory mediators can facilitate vasodilation and vascular leak, causing mucosal edema that can manifest clinically as nasal congestion and rhinorrhea in the case of inhaled allergens or gastrointestinal upset and diarrhea in the setting of food allergens [2]. Between 4 and 12 hours after initial exposure, a later phase response may potentially ensue with altered profiles of cellular adhesion markers including E-selectin and vascular cell adhesion molecules as well as chemoattractants including IL-5 facilitating the recruitment of eosinophils, basophils, T cells, and monocytes into the mucosa, leading to more persistent inflammation, epithelial damage, and mucosal edema [9, 10].

## Immunology of subcutaneous and sublingual immunotherapy

Much of our understanding of the mechanisms of AIT comes from studies in subcutaneous immunotherapy (SCIT), which has been in practice for over a century [11]. AIT influences allergen sensitivity by modulating aspects of the early and later phase responses of allergic inflammation [12]. SCIT has seen wide usage in management of airborne and insect venom allergies. However, use of SCIT in oral allergen therapy has been limited due to side effects [13].

During the repetitive allergen exposures of SCIT, APCs uptake allergen and travel to draining LNs where they induce regulatory T cells (Tregs), which secrete IL-10 and TGF-β, which tend to curtail Th2-mediated immunity in allergic inflammation [14–17]. SCIT can abrogate allergen-specific Th2 cells (Th2A), downregulate Th2 cytokines including IL-4, IL-5, and IL-13, and skew the immune response to Th1 cells [7, 18, 19].

The impact of SCIT on skewing T cell fates towards a regulatory phenotype facilitates the induction of B cell class switching with consequent production of allergen-specific IgG and IgA. These antibodies function to, among other possible mechanisms, compete with IgE for binding to allergen, preventing downstream FcεRI-mediated degranulation of mast cells and basophils and FcεRII (CD23)-mediated allergen presentation [12, 20, 21]. Generation of allergen-specific IgG with IgE inhibitory activity in the nasal mucosa has been demonstrated with strong associations to clinical response [22]. AIT is accompanied by an initial increase in allergen-specific IgE followed by a return to baseline levels or a drop below baseline over years [23–25]. SCIT can induce allergen-specific B cells, plasmablasts, and IL-10 and IL-1 receptor antagonist-producing B cells [22, 26]. Recent research has also demonstrated that SCIT can upregulate circulating follicular regulatory T cells (cTfrs) and downregulate circulating follicular helper T cells (cTfhs), which may ultimately contribute to suppression of IL-4 and IgE production [27, 28].

With regard to innate immune system modulation, SCIT has been implicated in curtailing the recruitment of basophils and eosinophils into the nasal epithelium [29, 30]. Notably, symptomatic improvement following SCIT was associated with diminished IL-5 expression and reduced recruitment of eosinophils into the nasal mucosa, likely as a reflection of diminished late phase reactions [31]. Furthermore, *ex vivo* studies of patients receiving SCIT have demonstrated suppression of basophil FcεRI-mediated activation and cytokine release, occurring in association with the upregulation of histamine receptor-2 [32].

In addition to its effects of basophils and eosinophils, SCIT was found to reduce skin mast cell populations, which in turn correlated with clinical response [33]. Furthermore, the induction of IL-10 in SCIT may mechanistically inhibit mast cell release of TNF-α, IL-8, and histamine [34].

More recent studies have uncovered an effect of SCIT on type 2 innate lymphoid cells (ILC2s). ILC2s, which secrete IL-5 and IL-13 to polarize naive T cells into Th2 cells [35, 36], appear to be downregulated by SCIT with concomitant expansion of Type 1 ILCs [37, 38].

Another mechanism by which SCIT elicits immunologic non-responsiveness may be through modulations in the composition of APCs. Specifically, SCIT can upregulate tolerogenic plasmacytoid dendritic cells (DCs) and CD141^+^ myeloid DCs and downregulate CD1c^+^ myeloid DCs [38]. SCIT has also been implicated in the induction of anti-inflammatory intermediate monocytes and downregulation of pro-inflammatory non-classical monocytes [38]. Taken as a whole, SCIT has been associated with restructuring of multiple compartments of the innate and adaptive immune systems, spanning initial antigen acquisition to the ultimate effectors of allergic inflammation.

As the immunologic mechanisms of SCIT have become clearer with recent studies, a key unknown that remains is the durability of protection conferred by SCIT. The ability of SCIT to induce a state of non-responsiveness to allergen even after the cessation of therapy has been demonstrated on some instances at least for a few years. A foundational randomized controlled trial (RCT) of participants treated with 3–4 years of grass SCIT demonstrated sustained improvements in allergic symptoms over at least 3 years after treatment discontinuation [39]. Furthermore, the participants who had discontinued SCIT had similar improvements in allergic symptoms compared to those who had continued SCIT. Immunologically, the participants who had discontinued SCIT exhibited diminished late skin responses and reductions in CD3^+^ T cell infiltrates and IL-4 expression on allergen challenge.

The duration of SCIT therapy appears to be an important factor in whether sustained non-responsiveness can be achieved [40]. Specifically, a 3-year duration of SCIT may be required to achieve some degree of sustained non-responsiveness after discontinuation of therapy [41]. Exploratory analyses of the GRASS RCT demonstrated that 2-years of SCIT therapy was unable to confer a sustained improvement in nasal symptom scores on allergen challenge at one year after SCIT discontinuation as compared to placebo [42]. Furthermore, differing allergens may be variably amenable to achieving sustained non-responsiveness. An RCT of 3-years of SCIT in ragweed allergy demonstrated a partial return of nasal reactivity on allergen challenge, suggesting that there may be some waning in protection over time [43]. Future studies exploring the persistence of key immunologic mechanisms of SCIT after discontinuation of therapy may elucidate the critical mediators of immunologic non-responsiveness.

Allergen provided by the sublingual route has been shown to be efficacious in reducing symptomatic rhinitis since the 1980s [44] and shares many mechanistic features with SCIT [45–50]. Sublingual immunotherapy (SLIT) has been widely used in therapy for aeroallergens including pollens and dust mite and has seen some initial promise in the treatment of peanut allergy [51]. SLIT relies on initial antigen capture by Langerhans cells (LCs) in the oral mucosa, followed by migration to draining LNs, including the submandibular and cervical chains, for antigen presentation to T cells [52–54]. This interaction stimulates generation of Th1 cells and Tregs while downregulating Th2 and Th2A cells, analogous to the mechanisms observed in SCIT [18]. Single-cell immunoprofiling implicated increased expression of musculin (MSC), a transcription factor that represses the activity of Th2 cells, in pathogenic Th2 cells among SLIT-treated patients one year into therapy [55]. Patients who had a favorable response to SLIT exhibited increases in MSC levels, raising its candidacy as a potential biomarker for clinical efficacy. Furthermore, SLIT is associated with regulatory changes at the epigenetic level with a possible induction of CD4^+^ CD25^high^ CD127^low^ CD45RO^+^ FOXP3^+^ memory Tregs with hypomethylation and increased expression of *Foxp3* [56]. SLIT has also been demonstrated to elicit a newly described subset of Tregs, namely IL-35–induced regulatory T cells (iTr35s), which further contribute to suppression of Th2 effector cells and cytokine production [57], although it is unclear if this mechanism is absent in SCIT.

*Ex vivo* analysis showed that both SCIT and SLIT can be associated with downregulation of cTfhs and upregulation of cTfrs, which in turn may prevent the induction of IL-4, IL-21, and IL-6 in the nasal mucosa after allergen exposure (Table 1) [58]. IL-4 is known to stimulate B cell class switching to IgE and IL-21 has more nuanced role in the regulation of IgE production [59, 60]. *Ex vivo* analysis further demonstrated significant differences in chromatin accessibility in cTfhs and cTfrs between SCIT and SLIT [58], the clinical impact of which remains to be elucidated.

**Table 1.** Clinical studies comparing immunologic correlates in SCIT and SLIT

Allergen	Study system	Immunologic correlates			Clinical response	References
		Humoral	Cell-mediated	Other		
HDM	RCT	↑ In sIgG_4_ in SCIT and SLIT groups (as compared to baseline)			Improvement in symptom scores for rhinitis and asthma and MS in SCIT group Improvement in rhinitis symptom scores and MS in SLIT group	Mungan [61]
Birch	Non-randomized, no control	= sIgE after pollen season in SCIT and SLIT groups ↑ In sIgG_4_ in only SCIT group (trend towards ↑ in SLIT group)			No difference in CSMS between SCIT and SLIT groups	Mauro [62]
HDM	Non-randomized, no control	↑ In sIgE and sIgG_4_ in only SCIT group ↓ In sIgE/sIgG_4_ in SCIT and SLIT groups	↑ In CD4^+^ CD25^+^ cells in only SCIT group		Improvement in subjective evaluations in SCIT and SLIT groups	Antúnez [63]
HDM	RCT	↓ In sIgE in SCIT and SLIT groups			Improvement in total symptom and MS in SCIT and SLIT groups	Eifan [64]
HDM	RCT	↓ In sIgE in SCIT and SLIT groups ↑ In sIgG_4_ in only SCIT group		↑ In serum IL-10 levels in SCIT and SLIT groups ↓ In nasal eosinophils after nasal provocation in SCIT and SLIT groups	Improvement in symptom scores and MS for rhinitis and asthma in SCIT group Trend towards improvement in symptom scores and MS for rhinitis and asthma in SLIT group	Yukselen [65]
HDM	RCT	Greater ↑ in sIgG_4_ in the SCIT group compared with SLIT group = tIgE and sIgE in SCIT and SLIT groups			Improvements in total symptom scores in both SCIT and SLIT groups	Karakoc-Aydiner [66]
Grass	RCT		↓ In frequencies of allergen-specific CD4^+^ T cells in SCIT and SLIT groups (though reversed 1 year after discontinuation) Greater ↓ in allergen-specific Th2 cells in SCIT group compared with SLIT group		= Total nasal symptom scores after allergen test in SCIT and SLIT groups (1 year after discontinuation of therapy, as reported in [42])	Renand [18]
Mono- or poly-sensitized to different allergens	Prospective comparative case series	↓ In tIgE in SCIT and SLIT groups			Improvement in total ocular symptom scores and MS in SCIT and SLIT groups	Sayed [67]
HDM	RCT	↑ In sIgG_4_ in SCIT and SLIT groups (higher levels in SCIT group)	Trend towards ↑ in CD4^+ ^CD25^+ ^FoxP3^+^ Tregs in SCIT and SLIT groups	↑ In serum interferon-γ only in SCIT group	Improvement of total rhinitis score and MS in SCIT and SLIT groups	Xian [68]
Grass	RCT	Greater ↑ in sIgA_1/2_ in SLIT compared to SCIT Greater ↑ in sIgG and sIgG_4_ in SCIT compared to SLIT			= Total nasal symptom scores after allergen test in SCIT and SLIT groups (1 year after discontinuation of therapy, as reported in [42])	Shamji [69]
Grass	Cross-sectional study		↓ In cTfh cells in SCIT and SLIT groups ↑ In Tfr and IL-10^+^ cTfh cells in SCIT and SLIT groups		Improvement in total symptom scores in SCIT and SLIT groups	Sharif [58]
Grass	SCIT cross-sectional study and SLIT RCT		Restoration of IL-10^+^ KLRG1^+^ ILC2s in both SCIT and SLIT with correlation of frequencies of IL-10^+^ ILC2s with symptomatic improvement		Improvement in total symptom scores in SLIT group (SCIT evaluation was cross-sectional)	Golebski [70]

In regards to the humoral response, clinical studies have demonstrated that SLIT can induce allergen-specific IgG4 and IgG2 production with corresponding increases in allergen-specific IgG4^+^ and IgG2^+^ memory B cells [46]. A recent RCT demonstrated that induction of allergen-specific IgG2 by SLIT was associated with favorable clinical responses [71]. An RCT comparing SCIT and SLIT demonstrated that SLIT generates more robust allergen-specific IgA responses, most prominently in nasal fluid, which may be expected based upon the mucosal route of delivery (Table 1) [69]. In contrast, SCIT generated a stronger allergen-specific IgG4 response than SLIT. Furthermore, in both SCIT and SLIT, the induction of a population of IL-10 secreting ILCs was associated with improved clinical response, possibly acting mechanistically through Th2 suppression and the maintenance of epithelial barriers against allergens [70]. A recent *ex vivo* analysis of patients with lipid transfer protein allergy has additionally demonstrated ILC modulation by SLIT [72].

Additionally, SLIT can have immunomodulatory effects at the level of APCs [73]. Specifically, SLIT can downregulate the costimulatory receptor CD86 and induce a subset of regulatory DCs, characterized by increased expression of complement component 1Q and stabilin-1 [74, 75]. Furthermore, the expression of complement 1Q and stabilin-1 in DCs was increased among patients who demonstrated a clinical response to SLIT as compared to nonresponders or placebo-treated patients, suggesting potential as a clinical biomarker.

Akin to SCIT, several studies have suggested that SLIT may be able to induce a persistent state of non-responsiveness to allergen, even after years of discontinuation of therapy. A number of RCTs analyzing 3-year courses of SLIT in grass pollen allergy have demonstrated sustained symptomatic control in the 1-2 year period after discontinuation of therapy [76–79]. In association with these clinical findings, SLIT-treated patients had persistent reductions in total IgE, allergen-specific IgE, and diminished skin test reactivity 2 years after treatment was discontinued [76]. Furthermore, another RCT evaluating patients at two years post-treatment demonstrated sustained increases in both allergen-specific IgG4 and in IgE-blocking activity [79]. As with SCIT, the duration of SLIT therapy appears to be critical in whether sustained non-responsiveness can be achieved. The GRASS RCT demonstrated that a 2-year course of SLIT for grass allergy was insufficient in improving nasal symptoms upon allergen challenge at 1-year post-therapy as compared to placebo [42]. Longer studies will be needed to ascertain the persistence of these effects as well as to explore the duration of protection in other systems beyond grass allergens.

Other methods of AIT were conceived to maximize practical aspects of treatment delivery and therapeutic longevity by shortening the duration of intervention while minimizing systemic allergic side effects [80]. The rationale for these approaches would be to deliver allergen to tissue containing abundant APCs to maximize immunogenicity while also containing few mast cells and limited vasculature to minimize both local and systemic reactions.

## Epicutaneous immunotherapy

The precedent for epicutaneous immunotherapy (EPIT) was set in the 1950s among patients who underwent needle scarification of the forearm with pollen extract, resulting in improvement in hay fever outcomes [81]. EPIT was revisited in the 2000s as an AIT modality that would capitalize on the high density of antigen-presenting LCs in the epidermis while minimizing risk of systemic reactions due to poor vascularity. Initial studies had utilized an adhesive tape stripping approach in EPIT to remove layers of stratum corneum, generating an inflammatory response from keratinocytes with increased LC expression of MHC class II, CD86, CD40, CD54, and CD11c, accompanied by migration to draining LNs [82]. The initial RCT of tape stripping EPIT for grass pollen allergy demonstrated significant symptomatic improvement [83].

The Epicutaneous Viaskin Patch is a more recent mode of delivery in EPIT which consists of a transparent plastic membrane loaded with powdered allergen, creating an occlusive chamber and utilizing transepidermal water loss to increase permeability of the stratum corneum to solubilize allergens and facilitate accessibility to APCs [84, 85]. Viaskin patch EPIT has shown significant promise in the treatment of peanut allergy [86]. However, Viaskin patches continue to have low rates of allergen delivery into the epidermis, suggesting potential for further optimization [85]. More recently, a powder-laden, dissolvable microneedle array (PLD-MNA) has emerged as an EPIT platform to optimize allergen delivery [87]. In this modality, microneedles loaded with powdered allergens are inserted into the skin. The shafts of the microneedles dissolve, releasing the powdered allergen, which is retained in the epidermis with minimal escape into the circulation [88].

In contrast to SCIT, EPIT has seen use in both oral allergens and aeroallergens (Table 2) [89]. A recent systematic review concluded that EPIT was likely efficacious in inducing non-responsiveness in peanut allergy with uncertain benefit for cow milk or grass pollen [90]. In EPIT using the Viaskin Patch, initial antigen acquisition relies on both CD11b^+^ CD64^+^ classical dendritic cells type 2 (cDC2s) and LCs [91, 92]. One murine model study demonstrated that PLD-MNA may induce skin-resident CD11b^+^ CD169^+^ macrophages to produce IL-10 and TGF-β at the immunization site, which could systemically potentiate Tregs in lymphoid tissue [87].

**Table 2.** Immunologic correlates of novel immunotherapy modalities in clinical studies

Modality	Allergen	Study system	Immunologic correlates			Clinical response	References
			Humoral	Cell-mediated	Other		
1. EPIT	Cow milk	RCT	= sIgE			No change in cumulative tolerated dose on food challenge at 3 months	Dupont [93]
	Peanut	RCT	= sIgE			Not evaluated	Jones [94]
	Peanut	RCT	↑ sIgG_4_, sIgG_4_/sIgE ratio = sIgE, tIgE, % sIgE			Improvement in successfully consumed dose on food challenge	Jones [95]
	Peanut	RCT	↑ sIgE followed by return to baseline ↑ sIgG_4_			Improvement in eliciting dose on food challenge	Sampson [96]
	Peanut	RCT	↑ sIgE followed by return to baseline ↑ sIgG_4_			Improvement in eliciting dose on food challenge	Fleischer [86]
	Peanut	Non-randomized, no control (open label extension)	↑ sIgG_4_ ↑ sIgE followed by return to baseline			Improvement in eliciting dose on food challenge	Fleischer [97]
	Peanut	RCT	↑ sIgG_4_		= Basophil activation tests	Improvement in successfully consumed dose on food challenge	Scurlock [98]
	Peanut	Non-randomized, no control (2 months of discontinuation of EPIT)			= sIgE, sIgG_4_	Continued non-responsiveness on food challenge	[99] Brown-Whitehorn 2021
	Peanut	RCT	Positive association between proportion of allergen-specific type 2 cells after allergen stimulation and sIgE levels	**↓** In CD154^+^ IL-4^+^ and CD154^+^ IL-13^+^ cells after allergen stimulation ↑ In CD154^+^ IL-10^+^ cells after allergen stimulation Negative association between proportion of allergen-specific type 2 cell and successfully completed dose on oral food challenge	Positive association between proportion of allergen-specific type 2 cells after allergen stimulation and basophil activation tests	As per Jones [95]	Berin [100]
2. Intradermal	Grass	Randomized, no placebo	↑ sIgG		**↓** Cutaneous late phase responses	Not evaluated	Rotiroti [101]
	Grass	RCT	↑ sIgE	Skin biopsy with ↑ T cell expression of Th2 surface marker CRTH2 and decreased expression of Th1 marker CXCR3 Microarray of cultured T cells demonstrating increased IL-5 expression.		No effect on CSMS	Slovick [102]
	HDM	Non-randomized, no control	↑ sIgG_4_			Improvement in total nasal symptom scores	Vieira-Hernández [103]
	Grass	RCT	= sIgG_4_ ↓ sIgE			Improvement in CSMS	Sola Martínez [104]
	HDM	Non-randomized, no control	↑ sIgG_4_		↑ IL-10	Improvement in total nasal symptom scores	Rondon [105]
3. Intralymphatic	Grass	RCT	**↓** sIgE			Improvement on NPT and subjective symptom scores	Senti [106]
	Cat	RCT	= sIgG_1_, sIgE ↑ sIgG_4_	**↑** IL-10 = IL-2, IL-4, IL-5, IL-17, or IFN-γ		Improvement on NPT	Senti [107]
	Grass	RCT	↑ sIgE ↑ sIgG_4_	**↓** IFN-γ Tendency towards **↑ **IL-10 = FOXP3, IL-4 expression	= Pollen-induced histamine release	No effect on CSMS	Witten [108]
	Grass, birch	RCT	= sIgG_4_ ↑ sIgE	↑ Serum expression of activation markers CD69 and CD98 on CD4^+^ T cells = Allergen-stimulated IL-10 secretion	= Number of leukocytes, eosinophils, neutrophils, basophils, monocytes, and lymphocytes **↓** In number of living leukocytes and epithelial cells in nasal lavage Tendency toward **↓** in IL-8 in nasal lavage	Improvement in symptom scores	Hylander [109]
	Grass, birch	RCT	= sIgE, IgG4 ↑ sIgG_4_ affinity in subset of patients who had improvement in nasal symptoms	= CD4^+^ CD25^+^ FoxP3^+^ cells		Improvement in symptom scores	Hylander [110]
	Cat	RCT	↑ tIgG, IgG_4_, IgG_2_ = sIgG_1_, sIgG_3_, sIgE			As reported in Senti [107]	Freiberger [111]
	Grass, birch	RCT	↑ sIgG_4_ Transient ↑ sIgE	↑ Memory T cells with increased proportion of CCR7^-^ effector memory T cells in LNs Increase in CCR5^+^ Th1 cells and CD4^+^ CD25^+^ Tregs in blood		Improvement on NPT	Hellkvist [112]
	Japanese cedar	RCT	↑ sIgE ↑ sIgG followed by decrease = IgG_4_	= Number of FoxP3^+^ Tregs or Bregs between AIT and placebo		No significant change in CSMS Improvement on NPT	Terada [113]
	Birch or timothy	RCT	= sIgE ↑ sIgG, sIgG_4_			No significant change in NPT Improvement in MS	Konradsen [114]
	Mountain cedar	RCT	↑ sIgE (though similar ↑ in placebo)			Improvement in CSMS	Thompson [115]
	Grass	RCT	↑ sIgG_4_			No significant change in symptom scores	Weinfeld [116]
	Human dust mite, cat, dog	RCT			Trend towards **↓** percentages of activated CD63^+^ basophils on basophil reactivity test	No significant change in symptom scores or MS	Park [117]
	Grass	RCT	↑ sIgE ↑ sIgG_4_			Improvement in CSMS	Skaarup [118]
	Grass pollen	Study 1: RCT (previous grass SCIT) Study 2: RCT (no previous AIT)	↑ sIgG_4_ ↑ sIgE	↑ Intralymphatic DC expression of costimulatory molecules CD80 and CD86 = CCR5^+^ central memory T cells or CD25^++^ CD4^+^ effector memory T cells in blood		Study 1: improvement in CSMS Study 2: no improvement in CSMS	Hellkvist [119]
	Birch, grass pollen	RCT	**↓** sIgE = sIgG_4_	↑ Allergen-induced IL-10 secretion ↑ CD4^dim^ CD25^hi^ FoxP3+ Tregs and activated CD3^+^ CD4^+^ CD45RA^−^ FoxP3^++^ Tregs = resting CD3^+^ CD4^+^ CD45RA^+^ FoxP3^+^ Tregs		Improvement in symptom scores and MS	Ahlbeck [120]
	Bee venom	Study 1: non-randomized, no control Study 2: randomized, no control	Study 1: ↑ sIgG_1_ = sIgG_4_, sIgE Study 2: ↑ sIgG_,_ sIgE			Study 1: protection on insect venom challenge Study 2: several serious adverse effects on insect venom challenge	Chabot [121]
	Birch, grass	RCT	↑ sIgG_4_ **↓** sIgE	↑ CD4^+^ memory T cells in LNs	**↓** FcεR1 on basophils	Improvements in CSMS and MS No significant change in NPT	Hjalmarsson [122]

Following antigen acquisition, DCs and LCs present antigen to naïve CD4 T cells, triggering a cascade of skewed T cell fates comparable to what has been observed in SCIT and SLIT. An *ex vivo* analysis among patients following EPIT therapy demonstrated a reduction in the frequencies of allergen-specific type 2 cells, specifically CD154^+^ IL4^+^ and CD154^+^ IL-13^+^ cells and an increase in the frequency of CD154^+^ IL10^+^ cells [100]. In hallmarking a shift towards a regulatory phenotype, EPIT has been associated with an induction of CD25^+^ Foxp3^+^ CD4 T cells in lymphoid tissue [87, 123, 124]. Use of an anti-CD25 antibody was able to reverse induction of Tregs with consequent eosinophilic infiltration of the esophagus in peanut-sensitized mice, highlighting the role of Tregs in establishing peripheral non-responsiveness [125]. The shift towards a regulatory phenotype may be related to alterations at the level of APCs with a recent murine model study demonstrating that over the course of EPIT therapy, LCs gradually lost the ability to prime CD4^+^ T effector cells, instead developing the capacity to stimulate Tregs [126].

While induction of Tregs appears to be shared among AIT platforms, EPIT appears to induce unique Treg populations, potentially as a reflection of aspects of its mode of delivery. In a study comparing oral immunotherapy (OIT), SLIT, and EPIT, all modalities were able to induce effector/memory (Foxp3^+^ CD44^hi^ CD62L^-^) Tregs in peanut-sensitive mice [127]. However, only EPIT was able to additionally induce naïve (Foxp3^+^ CD44^lo^ CD62L^+^) Tregs. Underlying the significance of this mechanism, 8 weeks after discontinuation of AIT, only the Tregs induced by EPIT demonstrated persistent immunosuppressive activity in contrast to those induced by SLIT or OIT. This long-lasting protection may have clinical correlates in establishing a state of non-responsiveness to allergens, whereby patients remain desensitized despite discontinuation of AIT. Promisingly, two initial studies of patients with peanut allergy who had been treated with long-term EPIT and then discontinued for 2 months demonstrated transient non-responsiveness of peanut after treatment [97, 99]. Of note, the duration of protection after discontinuation of therapy demonstrated in this setting is limited in comparison to the long-term protection conferred by SCIT and SLIT in grass pollen allergy.

Another murine model study highlighted the significance of induction of CD4^+^ CD25^+^ Foxp3^+^ CD62L^+^ Tregs in the ‘bystander effect’ whereby transfer of CD62L^+^ Tregs induced by milk-EPIT allowed for protection against sensitization to a separate antigen, peanut [128]. The bystander effect was hypothesized to be linked to the hypermethylation of the *Gata3* promoter, involved in Th2 differentiation, with consequent downregulation of Th2 cytokines including IL-4, IL-5, and IL-13, as well as hypomethylation and upregulation of *Foxp3*. These epigenetic modulations are also hypothesized to mechanistically influence the durable clinical protection conferred by EPIT [99].

Induction of intestinal non-responsiveness to food allergens may require Tregs with the capacity to home to intestinal tissue to undergo local proliferation in the intestinal lamina propria [129]. Certain LN distributions within the gut may be predisposed to generate tolerogenic versus pro-inflammatory immune responses [130]. In a murine model, drainage of dietary antigens into the proximal intestine was associated with FOXP3^+^ Treg-mediated immunity, whereas delivery of antigen to the LNs in the distal GI tract resulted in generation of effector T cells. These findings suggested that compartmentalized delivery of antigens may be a potential strategy to influence tolerogenic immunity.

Insight into how EPIT can induce gut-specific non-responsiveness to oral allergens was elucidated in a set of recent studies [91, 92, 131]. Firstly, in a comparison of EPIT and OIT in the prevention of ovalbumin (OVA)-induced anaphylaxis in mice models, only EPIT demonstrated protection four weeks after discontinuation of therapy [131]. EPIT induced a special population of Tregs, namely Foxp3^-^ latency-associated peptide (LAP)^+^ Tregs, which functioned to directly inhibit mast cell function in a TGF-β dependent mechanism. Furthermore, while T cells activated in cutaneous LNs generally exhibit homing towards cutaneous tissue, the LAP^+^ Tregs induced by EPIT demonstrate homing capacity for both the skin (CCR4) and gut (CCR6, CCR9) [131, 132]. The LAP^+^ Tregs appear to be transiently activated, appearing after 2 weeks of EPIT though not persisting after 8 weeks [92]. These findings led to the suggestion that induction of LAP^+^ Tregs may be part of the initial mechanistic induction of EPIT, followed by a more persistent response mediated by CD62^+^ Tregs [123]. The efficacy of EPIT in oral allergen therapy highlights the interconnectedness of the immune system, whereby antigen exposure in the skin can induce sustained and clinically relevant alterations in the gut mucosa.

The potential evolutionary rationale for the skin-mucosal surface immune connection uncovered with the use of EPIT is intriguing. There is a strong precedent for skin epithelial barrier dysfunction driving allergic sensitization as illustrated by the atopic march, hallmarked by disease progression from atopic dermatitis to allergic rhinitis, asthma, and food allergies, driven by pathophysiology whereby local inflammatory responses facilitate barrier breakdown in distant organs, including the gastrointestinal and respiratory tracts [133]. In a paralleled fashion that underlies the pattern of immune dysregulation in the atopic march, EPIT appears to harness skin-gut immune mechanisms, namely unique tissue-homing markers, to transduce stimuli at the cutaneous level to establish non-responsiveness in the gut.

In regards to the humoral response to EPIT, clinical trials have demonstrated elevations in allergen-specific IgG4 [86, 95, 96]. However, the significance of the humoral response is uncertain with one study demonstrating that passive sensitization of naïve mice with sera from EPIT-treated mice was ineffective in preventing anaphylaxis, arguing against systemically accessible IgE-blocking IgG antibodies as the sole mechanism of non-responsiveness to oral allergens in EPIT [131]. Consistent with SCIT, EPIT has been shown to induce initial elevations in allergen-specific IgE followed by return to baseline levels in clinical studies [86, 96].

## Intradermal immunotherapy

The precedent for intradermal immunotherapy (IDIT) was established by Phillips in 1926 who reported symptomatic improvement with IDIT in a group of 29 patients with grass pollen allergy [134]. IDIT has since seen use in both aeroallergens and insect venom therapy (Table 2). An *ex vivo* study of human skin demonstrated that initial antigen acquisition in IDIT is most efficiently achieved by CD14^+^ dermal DCs, followed by CD1a^+^ dermal DCs [135]. Another murine model study identified a significant role of Langerin^+^ dermal DCs in mediating the immunoregulatory impacts of IDIT [136]. IDIT, as compared to SLIT or EPIT, resulted in high allergen-specific IgG production with reductions in allergen-specific IgE [136]. Notably, upon experimental deletion of Langerin+ dermal DCs, there was a restoration of allergen-specific IgE production, highlighting the likely significance of these APCs in mediating the response to IDIT therapy.

Despite promising initial findings [101], the first RCT evaluating IDIT in grass pollen failed to demonstrate clear clinical improvements [102]. Immunologic biomarkers demonstrated an increase in allergen-specific IgE and cutaneous biopsies revealed increased expression of Th2 surface marker CRTH2 and decreased expression of Th1 marker CXCR3. However, a later RCT using IDIT in grass pollen did demonstrate symptomatic improvements with an accompanying reduction in allergen-specific IgE and no changes in allergen-specific IgG4 [104]. Other studies of IDIT in house dust mite (HDM) allergy were suggestive of clinical efficacy and demonstrated increases in allergen-specific IgG4 and IL-10 [103, 105]. These findings, taken as a whole, suggest that while IDIT may rely on unique APCs for antigen acquisition and presentation, the mechanisms of allergen-specific IgG generation and the shift towards regulatory cytokine production in IDIT appear to be shared with SCIT and SLIT.

To circumvent the side effects and risk of IgE-mediated reactions that can accompany administration of whole allergen, peptide AIT was conceptualized. Peptide AIT utilizes either short peptides or long contiguous overlapping peptides, featuring key CD4 T cell epitopes of allergens while lacking the conformation of the intact allergen to prevent IgE recognition [137]. Peptide AIT is often administered subcutaneously or intradermally and has been recently reviewed elsewhere [137, 138].

## Intralymphatic immunotherapy

In intralymphatic immunotherapy (ILIT), aeroallergens are generally administered to the inguinal LNs to induce a strong T-cell response while requiring smaller amounts of allergen and reduced treatment durations (~2 months) [139, 140]. As compared to SCIT, ILIT appears to cause fewer adverse effects and improves patient compliance [106]. In a murine model study comparing SCIT and ILIT, ILIT generated greater than 10-fold higher allergen-specific IgG2a response despite requiring 100-fold lower allergen doses than SCIT [141]. Biodistribution evaluations confirmed that ILIT had superior antigen delivery and retention in the LNs, whereas SCIT delivered a significant fraction of antigen to the liver. The lower allergen doses required in ILIT correlate with robust safety profiles in multiple meta-analyses of ILIT [139, 142]. Furthermore, a murine model study comparing immune responses to subcutaneous, intradermal, intramuscular, and intralymphatic routes of administration suggested that only the intralymphatic route was able to elicit a robust IgG2a response as well as Th1-skewed response with increased IFN-γ production [143]. In contrast, all routes of administration were able to induce a robust IgG1 response.

In clinical settings, ILIT administered in 4-week intervals as three doses has shown comparable symptomatic improvements to a 3-year course of SCIT with similar decreases in allergen-specific IgE [106]. In a study where ILIT was administered over 2-week intervals as opposed to 4-week intervals, there was no evidence of symptomatic improvement despite increases in allergen-specific IgG4 and a tendency towards higher IL-10 levels [108]. It was suggested that the 4-week interval may be important in the success of ILIT due to improved memory B-cell development and antibody affinity maturation [80]. Notably, under the 4-week intervals, there may have been periods of limited antigen availability in the lymphatic follicles, which could influence competitive selection of high-affinity memory B cells.

ILIT likely operates through similar humoral and cell-mediated mechanisms as SCIT and SLIT. One of the initial RCTs of ILIT demonstrated induction of allergen-specific IgG4 and IL-10 [107]. ILIT-mediated generation of allergen-specific IgG4 has since been recapitulated in multiple studies [111, 112, 116, 118, 144]. However, the effect of ILIT on allergen-specific IgE levels appeared unclear in a recent meta-analysis [144]. Furthermore, ILIT may induce immunologic changes in target nasopharyngeal tissues with one murine model study of ILIT demonstrating decreased eosinophil counts and reduced mRNA expression of IL-4, IL-6, IL-17A, and IL-33 in nasal mucosa [145]. Diminished eosinophil recruitment in ILIT appears to be mediated by reduced expression of the chemokine eotaxin-2, while diminished neutrophil recruitment may be related to reduced expression of CXCL1 and CXCL2 [146].

A recent murine model study revealed that ILIT can diminish the production of serum Th2 cytokines including IL-13, IL-25, and IL-33, consistent with the Th2 dampening seen across AIT modalities [145]. However, in contrast to the Th1 skewing observed in many AIT modalities, a number of ILIT studies have found evidence of ILIT-induced downregulation of Th1 and Th17 pathways [145, 146].

In an RCT, ILIT appeared to increase levels of CD4^+^ CD25^+^ effector memory Tregs in the peripheral blood, consistent with shifts towards regulatory immunity [112]. Furthermore, ILIT can induce increases in effector memory T cells within the LNs, suggesting that some of the immunoregulatory effects of ILIT can be mirrored in both the blood and LNs [112, 122].

Some studies did not demonstrate that ILIT was able to specifically induce FOXP3^+^ Tregs [110, 113]. However, a recent murine model study did demonstrate significantly higher levels of FOXP3^+^ Tregs following ILIT [145]. A recent study gave evidence of the ‘bystander effect’ in ILIT. In this regard, patients sensitized to both birch and grass pollen had received ILIT for either birch or grass and then demonstrated symptomatic improvements not only during the months of the targeted allergen season but also during the non-targeted allergen season [120]. This finding occurred in the setting of increases in IL-10 secretion and upregulation of CD4^dim^ CD25^hi^ FoxP3^+^ Tregs and activated CD3^+^ CD4^+^ CD45RA^−^ FoxP3^++^ Tregs. This transition to a regulatory phenotype was hypothesized to confer a dampening of overall allergic inflammation, occurring independently of the triggering allergen, comparable to the bystander phenotypes described in EPIT and SCIT [128, 147, 148].

Another study demonstrated that ILIT-induced CD3^+^ CD4^+^ Foxp3^+^ Treg cells with increased expression of CCR7, a LN homing marker, suggestive of increased propensity for Treg recirculation in lymphoid tissues [149], which may be expected to suppress both new and recall lymphocyte reactions to allergen. Furthermore, studies have shown decreased basophil FcεR1 expression and a trend towards diminished basophil reactivity, highlighting potential modulation at the level of the innate immune system [117, 122]. As a whole, multiple recent systematic reviews have concluded that ILIT appears to be safe in treatment of allergic rhinitis though with widely varying reports of long-term efficacy in setting of significant trial heterogeneity [139, 142, 144].

## Conclusion

Allergen contact appears to be required for some individuals to develop hypersensitivity to that allergen. Paradoxically, allergen exposure, presumably processed through similar pathways of tissue and immune interactions, is also a path for desensitization. The different outcomes of allergen-mediated sensitization versus desensitization by contact with the same allergen may have at their root differences in allergen dosage, frequency, context, host factors, adjuvant availability, and route through which allergen gains access to the body. In this light, although different routes may have different propensities for context-dependent immune responses, the fact that each of the diverse routes of allergen entry reviewed here can achieve desensitization is consistent with a model that sensitization may also not depend upon a particular entry portal. Even aerosolized allergen delivered through the nasal route, a likely route for pollen sensitization, can lead to allergen-specific desensitization [150–152]. In addition, pollen delivery to the gastrointestinal tract can desensitize to aeroallergens in the case of birch [153, 154] and possibly ragweed [155, 156], consistent with a model of cross-system induction of immunologic non-responsiveness to allergens.

Immune system analysis in controlled desensitization experiments illuminates a general structure of antigen/allergen processing in different barrier tissues. In general, there is indirect or direct antigen delivery to LNs, followed by upregulation of Tregs to establish peripheral non-responsiveness to allergen. The immunologic differences between AIT routes reside in which APCs facilitate antigen acquisition, their physiologic state/inclination, and aspects of their movement. Other differences include the kinetics of antigen presentation in LNs, the allergen-specific Ig responses generated, and potentially, the type of Treg induced, which may be connected to variation in tissue-specific homing properties.

Regarding this latter point, a connection between skin and gut is illuminated by the efficacy of EPIT in the treatment of peanut allergy, perhaps related mechanistically by the induction of gut-homing LAP^+^ Tregs as well as long-lived naïve CD62L^+^ Tregs. Meanwhile, in ILIT, the induced Treg population harbors homing markers for LNs, facilitating continued recirculation through secondary lymphoid organs, potentially influencing the anatomic reach and longevity of allergen-specific—and perhaps bystander allergen—non-responsiveness. Important questions that emerge from mechanistic considerations of desensitization is what do these regulatory pathways look like during the allergen sensitization phase, and what are the mechanisms that shift the balance instead towards intolerance. Ongoing work promises to lead to deeper insights into these questions and other long-standing puzzles of allergic disease. In addition, a more complete understanding of the mechanisms that facilitate the persistence of immunologic non-responsiveness to allergen after discontinuation of immunotherapy will be vital in maximizing the clinical impact of AIT.

## Data Availability

No new data were generated or analyzed in support of this manuscript.

## Abbreviations

AIT: allergen immunotherapy
APCs: antigen-presenting cells
BAL: bronchoalveolar lavage
Breg: regulatory B cell
cTfh: circulating follicular helper T cell
cTfr: circulating follicular regulatory T cell
CSMS: combined symptoms and medications score
DCreg: regulatory dendritic cell
EPIT: epicutaneous immunotherapy
HDM: house dust mite
IDIT: intradermal immunotherapy
ILC: innate lymphoid cell
ILIT: intralymphatic immunotherapy
iTr35: IL-35–induced regulatory T cells
LAP: latency-associated peptide
LC: Langerhans cell
LN: lymph node
MS: medication scores
MSC: musculin
NPT: nasal provocation testing
OIT: oral immunotherapy
OVA: ovalbumin
PBMC: peripheral blood mononuclear cell
RCT: randomized controlled trial
SCIT: subcutaneous immunotherapy
sIg: allergen-specific immunoglobulin
Th2A: allergen-specific Th2 cell
tIg: total immunoglobulin
Treg: regulatory T cell
