# Correction to: Immunology of allergen immunotherapy

**DOI:** 10.1093/immadv/ltad017

**Published:** 2023-10-12

This is a correction to: Rifat S Rahman, Duane R Wesemann, Immunology of allergen immunotherapy, Immunotherapy Advances, Volume 2, Issue 1, 2022, ltac022, https://doi.org/10.1093/immadv/ltac022

The Conflict of interest statement of this manuscript has been corrected.

